# Incidence of infectious endophthalmitis after keratoplasty surgery: an updated systematic review and meta-analysis

**DOI:** 10.1007/s15010-026-02772-4

**Published:** 2026-04-17

**Authors:** Kai-Yang Chen, Hoi-Chun Chan, Chi-Ming Chan

**Affiliations:** 1 Department of General Medicine, Chang Gung Memorial Hospital (Linkou Branch), Taoyuan, Taiwan; 2 School of Pharmacy, China Medical University, Taichung, Taiwan; 3 Department of Ophthalmology, Cardinal Tien Hospital, New Taipei City, Taiwan; 4 School of Medicine, Fu Jen Catholic University, New Taipei City, Taiwan

**Keywords:** Infectious endophthalmitis, Keratoplasty, Corneal transplantation, Postoperative complications, Incidence and risk factors, Penetrating keratoplasty, Endothelial keratoplasty

## Abstract

**Introduction:**

Infectious endophthalmitis is a rare but vision-threatening complication following keratoplasty. With the evolution of corneal transplantation techniques toward endothelial keratoplasty (EK) and anterior lamellar keratoplasty (ALK), contemporary pooled incidence estimates stratified by surgical technique and geographic region are required to inform perioperative prevention strategies and postoperative surveillance. This study aimed to provide an updated systematic review and single-arm meta-analysis evaluating the incidence and risk factors of infectious endophthalmitis after keratoplasty.

**Methods:**

This systematic review and meta-analysis was conducted according to PRISMA guidelines. Five databases (PubMed, Scopus, Web of Science, ScienceDirect, and Cochrane Library) were searched from inception to December 31, 2025. Observational studies reporting incidence of infectious endophthalmitis after keratoplasty were included. A random-effects single-arm meta-analysis of proportions with logit transformation was performed. Subgroup analyses were conducted by keratoplasty type, geographic region, study period, and follow-up duration. Heterogeneity was assessed using the I^2^ statistic and τ^2^, and prediction intervals (PIs) were calculated when appropriate.

**Results:**

Twenty-one studies comprising multiple keratoplasty procedures were included. The pooled incidence of infectious endophthalmitis after keratoplasty was 0.40% (95% CI 0.30–0.50), with a prediction interval of 0.08–1.95%, indicating substantial between-study variability. Significant heterogeneity was observed (I^2^ = 95.75%, τ^2^ = 0.59, p < 0.001). Stratified analyses showed higher incidence after penetrating keratoplasty (0.50%; 95% CI 0.30–0.60) compared with ALK (0.20%; 95% CI 0.01–0.30) and EK (0.20%; 95% CI 0.20–0.30) (p < 0.0001). By region, incidence was 0.40% in North America, 0.30% in Asia, and 0.70% in Europe (p = 0.007). Incidence varied by follow-up duration: 0.20% (≤ 1 month), 0.60% (1–12 months), and 0.30% (> 12 months) (p = 0.007). Frequently reported risk factors included penetrating keratoplasty, combined intraocular procedures, anterior vitrectomy, donor-related contamination, delayed suture removal, and higher systemic comorbidity burden. Visual outcomes were generally poor, with frequent graft failure and limited visual recovery.

**Conclusion:**

Post-keratoplasty infectious endophthalmitis remains uncommon but clinically consequential. Penetrating keratoplasty showed a higher pooled incidence than lamellar techniques in this synthesis, and meaningful geographic variation persists. These findings indicate lower pooled incidence estimates with lamellar techniques in this synthesis and highlight the importance of rigorous perioperative infection-prevention strategies.

**Supplementary Information:**

The online version contains supplementary material available at 10.1007/s15010-026-02772-4.

## Introduction

Endophthalmitis is an inflammatory process involving the intraocular cavities, particularly the anterior chamber and vitreous cavity [1]. This intraocular infection is characterized by high potential of ocular morbidity, including irreversible severe vision loss and, in some cases, loss of the eye [2]. One of the worst outcomes of intraocular surgery is postoperative endophthalmitis [3]. Although the general frequency of postoperative endophthalmitis has been decreasing since the late twentieth century, the reported incidence differs depending on the particular surgical operation and between research [4]. Incidences of endophthalmitis after various treatments range from 0.07 to 0.32% for cataracts [4–7], 0.03 to 0.07% for vitrectomy [8, 9], and less than 1% for posttraumatic patients with open globe injuries [10]. On the other hand, Levin et al. noted that only half of the patients after post-cataract surgery endophthalmitis achieved vision of 20/40 [11]. Similarly, Conrady and colleagues note that only 30% of patients regained a final visual acuity of 20/40 or better, whereas 31.2% had poor visual outcomes of count fingers or worse post-cataract surgery endophthalmitis [12].

Endophthalmitis has been categorized by clinical setting and time of onset of clinically apparent inflammation into four classifications: (1) posttraumatic (inflammation caused after a penetrating injury to the globe), (2) endogenous (inflammation caused from hematogenous spread of organisms to the eye from a distant anatomical site), (3) miscellaneous (inflammation resulting from spread of organisms from neighboring structures, secondary to microbial keratitis), and (4) postoperative (as a result of intraocular surgery) [13, 14]. Based on the mode of entry of the organism (mode of infection), endophthalmitis is classified as either endogenous or exogenous [15]. The majority of endophthalmitis cases are exogenous, meaning that organisms enter the eye via an infected cornea, trauma, or surgery. When the eye is seeded via the circulation, endogenous endophthalmitis develops. Though sometimes they solely exhibit ocular symptoms, patients often experience symptoms from their underlying systemic illness [16]. These types or categories of endophthalmitis determine the visual outcome, microbiology, and clinical presentation. The majority of endophthalmitis patients have acute symptoms that appear within hours to a few days. Since a delay in treatment might cause irreversible visual loss, these situations constitute medical emergencies.

A systematic review by Taban et al. analyzed 66 studies from 1963 to 2003, reporting a pooled incidence of 0.382% for acute endophthalmitis following penetrating keratoplasty (PK), with a noted decline over time—dropping from 0.453% in the 1990s to 0.200% by the early 2000s—attributed to advancements in surgical technique and postoperative care [17]. However, this review is now two decades old. The review does not reflect the current landscape, which includes widespread use of newer keratoplasty techniques like endothelial keratoplasty (EK) and anterior lamellar keratoplasty (ALK), as well as improved understanding of patient and regional risk factors. Therefore, there is a need for an updated systematic review and single-arm meta-analysis to assess the incidence of infectious endophthalmitis after keratoplasty, compare incidences across surgical types, and identify contemporary risk factors and outcomes to guide current clinical practice. The meta-analysis will summarize the available data, produce more accurate pooled incidences, and pinpoint variables (factors) linked to poor visual prognosis. The results are directly relevant to ophthalmologists in optimizing follow-up and care routines, initiating early interventions, and setting reasonable expectations in endophthalmitis patients. The primary outcome of the study was the incidence of endophthalmitis following keratoplasty, while the secondary outcomes were keratoplasty-related complications and risk factors contributing to endophthalmitis.

## Methodology

### Study design

This study is an updated systematic review and single-arm meta-analysis conducted in accordance with the PRISMA (Preferred Reporting Items for Systematic Reviews and Meta-Analyses) guidelines [18]. Our systematic review has been registered on an online registration website, PROSPERO, under ID number CRD420251065814.

### Search strategy

A comprehensive literature search was conducted using five major databases: PubMed, Scopus, Web of Science, ScienceDirect, and the Cochrane Library. The search covered studies published from inception to December 31st, 2025. The search strategy combined MeSH and free-text terms using Boolean operators: ("endophthalmitis" MeSH OR "endophthalmitis") AND ("penetrating keratoplasty" OR "Keratoplasty" MeSH OR "endothelial keratoplasty" OR "anterior lamellar keratoplasty" OR "deep anterior lamellar keratoplasty" OR "DALK" OR "Descemet stripping automated endothelial keratoplasty" OR "DSAEK" OR "Descemet membrane endothelial keratoplasty" OR "DMEK" OR "posterior lamellar keratoplasty" OR "corneal transplant" OR "corneal transplantation") AND "postoperative complications." A complete search strategy for each database is provided in the supplementary file (Table S1). Additionally, the reference lists of included studies were manually screened to identify any additional relevant articles.

### Study selection

Two reviewers (K.Y.C. and C.M.C.) independently screened titles and abstracts for relevance. Full-text articles were obtained and assessed for eligibility based on predefined inclusion and exclusion criteria. Any disagreements during the selection process were resolved by discussion and consensus with a third reviewer.

### Eligibility criteria

#### Inclusion criteria

Patients who underwent any type of keratoplasty surgery, including PK, EK, or ALK, regardless of age, sex, or underlying condition.

Studies evaluating keratoplasty procedures as the primary intervention, performed for any clinical indication.

Studies reporting original data on the incidence of infectious endophthalmitis after keratoplasty.

Studies involving human subjects who underwent corneal transplantation.

Observational studies, including retrospective or prospective cohort studies and case series, that mention incidence.

Studies published in English.

#### Exclusion criteria

Case reports, editorials, reviews, and conference abstracts without complete or extractable data were excluded.

Case series that do not focus on incidence or lack clear reporting of endophthalmitis rates.

Duplicate studies or studies with overlapping patient populations (the most comprehensive or recent study was included).

### Data extraction

A standardized data extraction form was used to collect the following information from each included study: study identifier (first author and publication year), study period, study design, population type and number, number of endophthalmitis cases, causative microorganisms, risk factors, and results. The primary outcome of the study was the incidence of endophthalmitis following keratoplasty. The primary outcomes were expressed as proportion of endophthalmitis cases relative to the total number of PK, EK, and ALK procedures performed in each study. In contrast, the secondary outcomes were complications associated with keratoplasty and risk factors contributing to endophthalmitis incidence. Data extraction was performed independently by two reviewers in duplicate, and discrepancies were resolved by consensus.

### Quality assessment

The quality of the included studies was assessed using the Newcastle–Ottawa Scale (NOS) for cohort studies [19], which evaluates methodological quality in terms of selection, comparability, and outcome domains; the comparability domain was interpreted cautiously because most included studies were non-comparative incidence cohorts. Under selection, the appraisal is based on representativeness of the cohort, exposure ascertainment, selection of the non-exposed cohort, and demonstration of the outcome of interest, where each section is awarded a maximum of four stars. The comparability domain only appraises the comparability of the cohorts, where a maximum of two stars is allocated. The three variables appraised under the outcome are assessment of the outcome, adequacy of the follow-up for the outcomes to occur, and adequacy of the follow-up of the cohorts, whereby each is allocated a maximum of one star. Good quality was allocated if the study attained three or four stars in the selection domain, one or two stars in the comparability domain, and two or three stars in the outcome/exposure domain. A quality of a study was fair if it only attained two stars in the selection domain, one or two stars in the comparability domain, and two or three stars in the outcome/exposure domain. Conversely, the methodological quality of the study was poor if it had zero or one star in the selection domain, zero stars in the comparability domain, as well as zero or one star in the outcome/exposure domain. Two reviewers independently carried out the methodological quality assessment. Any discrepancies between the two were discussed and resolved with the help of a third reviewer.

### Statistical analysis

A random-effects meta-analysis of single-group proportions was performed to estimate the pooled incidence of infectious endophthalmitis after keratoplasty. For the primary overall analysis, one independent estimate per study was used whenever possible to avoid double counting when multiple keratoplasty subtypes were reported within the same study. Technique-specific estimates were synthesized separately in subgroup analyses. The proportions were transformed using the logit transformation before pooling and back-transformed for interpretation of the pooled results. Studies reporting zero events were retained in the analysis. However, a continuity correction of 0.5 for the numerator (events) and denominator (sample size) was applied to enable calculation of the logit transformation. This analysis was performed using the event rate and a random-effects model in Comprehensive Meta-Analysis (CMA) version 3.7. Pooled incidences were calculated with corresponding 95% confidence intervals (CIs). The CIs in CMA were calculated using the Clopper-Pearson exact binomial method. With enough studies (at least three studies) [20], 95% prediction intervals (PIs) were estimated to reflect the expected range of incidence in future comparable studies. Forest plots were generated to illustrate individual study estimates, confidence intervals, and the pooled effect size. Statistical heterogeneity was evaluated using the I^2^ statistic, with I^2^ > 50% indicating substantial heterogeneity. A p-value < 0.05 was considered statistically significant. Narrative synthesis was also conducted as a standard approach to summarize findings on risk factors and complications related to keratoplasty. It complemented the meta-analyses by integrating qualitative results across studies where appropriate.

### Subgroup analyses

We performed various subgroup analyses to determine variation of endophthalmitis incidence. First, we conducted a temporal subgroup analysis by publication year (pre-2005 versus post-2005). The temporal subgroup analysis was essential to reflect changes in trends in keratoplasty in the last two decades compared to the prior decades. Secondly, we performed a subgroup analysis by types of keratoplasty to evaluate the variations in incidences of endophthalmitis across PK, EK, and ALK. Besides, we also performed subgroup analysis based on follow-up period to classify the outcomes on time-window stratification. Lastly, we performed a subgroup analysis according to geographic region (Asia, Europe, and North America) for the included studies to determine potential differences in endophthalmitis incidence related to regional differences.

### Publication bias

We inspected the asymmetry of funnel plots to determine the possibility of publication bias among the included studies. Quantitatively, we used funnel plot inspection and Egger’s regression test as exploratory assessments of small-study effects; these findings were interpreted cautiously given the rarity of events and substantial between-study heterogeneity. Specifically, a p-value of less than 0.05 in Egger’s regression test was deemed suggestive of small-study effects.

### Leave-one-out sensitivity analysis and meta-regression

The authors conducted random-effects meta-regression evaluation to explore potential sources of between-study heterogeneity. Pre-specified covariates included geographic region, publication year, keratoplasty type, and follow-up duration. We reported regression coefficients, 95% CIs, and p-values.

We also conducted a leave-one-out sensitivity analysis to assess the robustness of the overall pooled incidence. We sequentially removed each study to explore the influence of individual studies on the overall incidence.

## Results

### Search outcomes

A total of 1499 search results were identified via database searches. Before the initial screening, we identified and eliminated 873 duplicates. The remaining 626 records were screened based on title and abstract screening, leading to the removal of 349 irrelevant studies. These records addressed postoperative inflammatory conditions other than infectious endophthalmitis following keratoplasty. Two hundred and seventy-seven articles were sought for retrieval, but Only 63 reports were retrieved for full-text eligibility assessment criteria evaluation. Of the 277 reports sought for retrieval, 214 were not retrieved. The full-text eligibility evaluation led to only 21 studies meeting inclusion criteria, while 42 full-text reports were excluded as follows: irrelevant outcomes to our study objective (18), did not focus on incidence or lacked clear reporting of endophthalmitis rates (14), did not evaluate keratoplasty surgery (6), and focused on animal subjects (4).

**Fig. 1:**
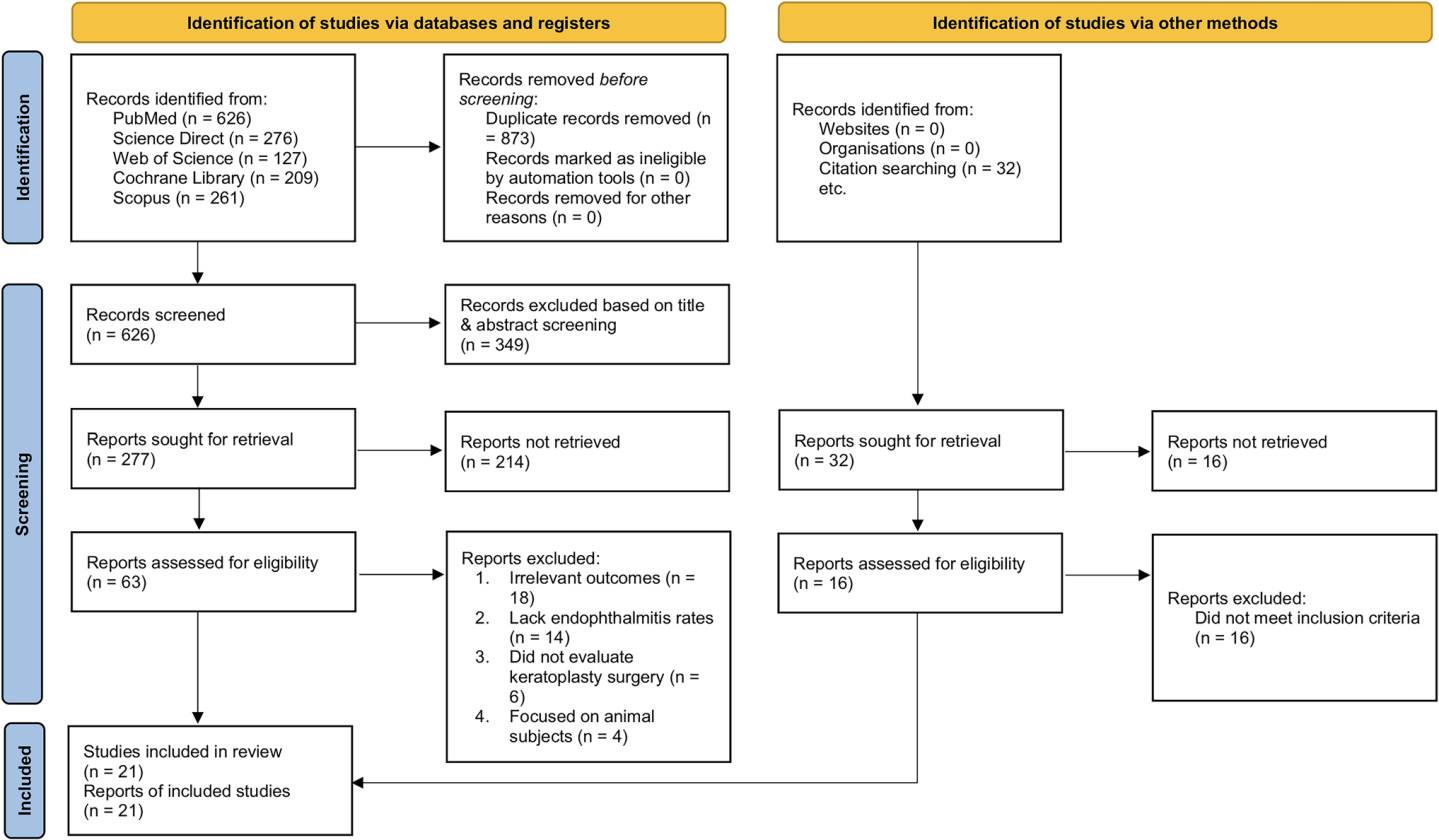
PRISMA 2020 flow diagram of the literature search, screening, eligibility assessment, and study inclusion process for the systematic review and meta-analysis

Additionally, 32 records were identified through other methods, specifically citation searching. Of these, 16 were not retrieved as they were duplicates, leading to the evaluation of 16 reports based on predefined eligibility criteria. However, all were excluded for not meeting the inclusion criteria. Supplementary Table S2 details the exclusion reasons for these articles that we eliminated for not meeting the inclusion criteria. In total, 21 studies were included in this review (Fig. 1).

### Characteristics of included studies

Table 1 below provides a summary of 21 studies that met the inclusion criteria. The included studies comprised 19 retrospective studies [4, 21–38] and two prospective studies [3, 39]. The study setting varied between Asia, Europe, and North America. The follow-up period among studies ranged from days (7–14 days) to years (20 years). Studies reported different complications that resulted from keratoplasty. Reported complications and adverse outcomes included poor visual outcomes, epithelial defects, donor rim contamination, severe visual loss, retinal detachment, and cystoid macular edema. Some of the risk factors that intensified the incidence of endophthalmitis were aphakia, donor rim culture contamination, organism resistance to therapy, and donor-related infection.

**Table 1:** Characteristics and results of the studies that met the inclusion criteria

Author ID	Study period	Study design	Setting	Population	Incidence of endophthalmitis	Causative microorganism	Follow-up (years)	Complications related to keratoplasty	Risk factors	Summary of findings/results
Guss et al. [21]	1980–1982	Retrospective case series	USA	445 PK cases	11/445 (2.47%)	Gram-positive bacteria: *Staphylococcus (*coagulase-positive & negative), *streptococcus pneumoniae,* alpha-hemolytic *streptococcus,* beta-hemolytic *streptococcus* Gram-negative bacteria: *Pseudomonas* aeruginosa Fungal: *Fusarium*	0.8	Poor visual outcomes, retinal detachment,	Aphakia, anterior vitrectomy	Despite treatment, final visual outcomes were poor
Leveille et al. [22]	1977–1982	Retrospective review	USA	1876 PK cases	4/1876 (0.213%)	Gram-positive bacteria: *Staphylococcus aureus*, *streptococcus pneumoniae,* group D *streptococcus-enterococcus* Gram-negative bacteria: *Pseudomonas* aeruginosa	2	Severe visual morbidity, persistent epithelial defect, aphakia	Positive donor rim cultures (strongly associated with infection), aphakia, topical corticosteroids	Eyes with positive donor rim cultures had a 22-fold increased incidence of endophthalmitis
Antonios et al. [23]	1983–1989	Retrospective review	USA	2210 PK cases	9/2210 (0.407%)	Gram-positive bacteria: *Staphylococcus aureus, streptococcus pneumoniae, streptococcus epidermidis* Fungal: *Candida albicans, torulopsis glabrata*	6	Severe visual loss, donor rim contamination, graft infection	Donor rim culture contamination	The risk of developing endophthalmitis is 12 times higher with a positive donor rim culture
Cameron et al. [24]	1983–1990	Retrospective review	USA	3000 PK cases	6/3000 (0.2%)	Gram-positive bacteria: *Staphylococcus aureus, enterococcus faecalis* Gram-negative bacteria: *Klebsiella pneumoniae* Fungal: *Candida albicans, torulopsis glabrata, aspergillus flavus*	2.25	Severe visual outcomes	Donor rim culture contamination, organism resistant to gentamicin	Donor rim cultures are significant in determining patients at increased risk of developing endophthalmitis
Kattan et al. [25]	1984–1989	Retrospective hospital-based survey	USA	1783 patients undergoing PK	2/1783 (0.112%)	Gram-negative bacteria: *Pseudomonas aeruginosa, klebsiella pneumoniae* Gram-positive bacteria: *Staphylococcus aureus, staphylococcus epidermidis, streptococcus viridans, streptococcus pneumoniae, streptococcus mitis, enterococcus faecalis*	5	Phthisis bulbi & corneal graft edema with poor vision (8/200)	Both corneoscleral rim cultures were negative; hence, no donor contamination	Only 2 cases of culture-proven endophthalmitis occurred in 1783 PK procedures
Aiello et al. [26]	1984–1987	Retrospective national outcomes study	USA	40,351 PK patients	310/40351 (0.768%)	None reported	2	Retinal detachment, Severe visual morbidity	Anterior vitrectomy (1.5 higher risk of endophthalmitis), Sex (males)	Patients undergoing PK had lower rates of retinal detachment but higher risk of endophthalmitis
Aaberg et al. [27]	1984–1994	Retrospective hospital-based incidence survey	USA	2805 patients with PK	5/2805 (0.178%)	Gram-positive bacteria: *Staphylococcus aureus, staphylococcus epidermidis, staphylococcus hominis, staphylococcus warneri, streptococcus viridans, streptococcus agalactiae, streptococcus mitis, Propionibacterium acnes, corynebacterium* Gram-negative bacteria: *Proteus mirabilis,* *Morganella morganii,* *Haemophilus influenzae,* *Pseudomonas aeruginosa,* *Serratia marcescens*	0.3 to 3	Poor vision, severe corneal edema, Pseudomonas infection, & retinal detachment	Donor cornea contamination in one case with positive donor culture	Visual acuity outcomes after treatment of endophthalmitis did not greatly improve in PK patients compared to those with secondary IOL implantation
Eifrig et al. [28]	1995–2001	Retrospective hospital-based incidence review	USA	2362 PK cases	2/2362 (0.085%)	Gram-positive bacteria: *Staphylococcus epidermidis, staphylococcus aureus, staphylococcus capitis, staphylococcus mitis, enterococcus faecalis* Gram-negative bacteria: *Serratia marcescens, proteus mirabilis, pseudomonas aeruginosa*	5.2	Severe vision loss	*Pseudomonas aeruginosa and Enterococcus faecalis* organisms	The 7-year incidence rate of acute-onset postoperative endophthalmitis was relatively lower
Keyhani et al. [29]	1998–2003	Retrospective review	USA	2466 PK cases	3/2466 (0.122%)	Fungal: *Candida albicans* *Candida glabrata* *Candida tropicalis*	7–14 days	Severe visual morbidity, poor penetration of antifungal prophylaxis, enucleation in some cases	Positive fungal donor rim culture contamination (strongly associated with infection)	The incidence of fungal endophthalmitis following PK were low, but they were associated with positive rim cultures
Jambulingam et al. [3]	2000–2007	Prospective study	India	1949 PK patients	10/1949 (0.513%)	Gram-positive bacteria: *Staphylococcus epidermidis, staphylococcus aureus, streptococcus *spp.*, enterococcus faecalis, micrococcus *spp.*, corynebacterium *spp.*, bacillus cereus, nocardia *spp. Gram-negative bacteria: *Pseudomonas aeruginosa, pseudomonas stutzeri, alkaligenes faecalis, klebsiella pneumoniae, acinetobacter* spp., c*itrobacter* spp., e*nterobacter* spp., f*lavobacterium* spp. Fungal: *Aspergillus flavus, candida albicans phialemonium dimorphosporum* Polymicrobial infections: *Micrococcus* spp. + *Acinetobacter* spp., *Enterobacter* spp. + *Trichosporon* spp., *Bacillus* spp. + *Enterococcus faecalis,* *Staphylococcus epidermidis* + *Acinetobacter* spp.	8	Severe visual loss in affected PK patients, Non-infectious inflammation, High culture-positive proportion donor rim correlation	Donor corneal rim contamination, fungal infection	Over an 8-year period, overall incidence of postoperative endophthalmitis was quite low
Wykoff et al. [30]	2002–2009	Retrospective	USA	2788 PK cases	3/2788 (0.108%)	Gram-positive bacteria: *Staphylococcus epidermidis, Staphylococcus aureus, Streptococcus pneumoniae, Streptococcus mitis, Streptococcus agalactiae*	3	Severe visual morbidity	Donor contamination	The frequency of acute -onset postoperative nosocomial endophthalmitis is low
Du et al. [38]	2006–2011	Retrospective cohort	USA	18,083 keratoplasty cases	190/18083 (1.05%)	None reported	0.5	None reported	Older age (≥ 75), male sex, immunosuppressant use, socioeconomic status	Corneal transplant had significantly risk of endophthalmitis than cataract surgery
Chen et al. [32]	1999–2006	Retrospective cohort	UK	PK: 11,320	PK: 76/11320 (0.671%)	Gram-positive bacteria: *Staphylococcus *spp. *Streptococcus *spp. *Mycobacterium *spp. Gram-negative bacteria: *Pseudomonas *spp. Fungal: *Candida spp.* *Fusarium *spp.	5	Severe morbidity (worsening of vision), reduced graft survival	Donor death due to infection, high-risk grafts, and therapeutic grafts	Affected patients with endophthalmitis had reduced graft survival and poor visual outcomes
Park et al. [31]	2004–2013	Retrospective review	South Korea	1594 PK cases	9/1594 (0.6%)	Gram-positive bacteria: *Staphylococcus epidermidis, staphylococcus aureus, staphylococcus capitis, staphylococcus mitis, enterococcus faecalis* Gram-negative bacteria: *Serratia marcescens, proteus mirabilis, pseudomonas aeruginosa*	3	Vitrectomy, retinal ischemia, retinal detachment, poor corrected visual acuity, cystoid macular edema	Choroidal detachment, suprachoroidal hemorrhage	Vitreoretinal complications after PK significantly occurred, leading to persistent reduction of corrected distance visual acuity
Borkar et al. [33]	2012–2018	Retrospective cohort study	USA	1676 PKs + 2292 EKs; 3968 patients	PK: 12/1676 (0.716%) EK: 4/2292 (0.174%)	Gram-positive bacteria: *Staphylococcus epidermidis,* *Methicillin-resistant Staphylococcus aureus (MRSA),* *Streptococcus discalcatiae,* *Streptococcus mitis,* *Enterococcus faecalis* Gram-negative bacteria: *Hemophilus influenzae,* *Serratia marcescens* Fungal *Candida *spp. *Colletotrichum truncatum*	2	Poor vision at three and six months Graft failure, retinal detachment, and cystoid macular edema	Anterior vitrectomy (OR 8.66; CI 2.98–25.18); PK more prone than EK	16 total endophthalmitis cases: 12 after PK, 4 after EK; 54.6% were female; VA outcomes significantly worse after PK-related infection (P = .01); higher graft failure in PK group (P = .02)
Das et al. [4]	2006–2018	Retrospective observational	India	6750 PKs 5315 EKs	EK: 18/5315 (0.34%) PK: 10/6750 (0.15%)	Gram-negative bacteria: *Pseudomonas aeruginosa,* *pseudomonas putida,* *Enterobacter cloacae, Burkholderia mallei,* *Klebsiella pneumoniae, Escherichia coli, Aeromonas hydrophila,* *Proteus mirabilis* Gram-positive bacteria: *Staphylococcus aureus*, Unidentified bacillus Fungal *Aspergillus flavus* *Aspergillus fumigatus* *Candida *spp.	0.75	Phthisis bulbi, poor visual outcome, failed graft	Donor-related infection, donor death causes	EK had a higher risk of endophthalmitis than PK
Anshu et al. [39]	1991–2010	Prospective cohort	Singapore	1206 PK patients	7/1206 (0.58%)	None reported	20	Optical grafts (endothelial failure, glaucoma), therapeutic/tectonic grafts (glaucoma)	Better survival (younger age) Higher donor endothelial cell count (better survival)	Graft survival reduced over time from 91 to 44%
Chen et al. [34]	2015–2019	Retrospective cohort	China	161 PK patients 94 ALK	PK: 5/161 (3.11%) ALK: 1/94 (1.06%)	Fungal: *Alternaria *spp. *Aspergillus *spp. *Fusarium *spp. *Candida *spp. *Pythium *spp. *Curvularia lunata* *non-spore-forming fungi*	2.8	PK group had higher risk of glaucoma, rejection, wound dehiscence, graft ulcers, and enucleation. ALK group had lower complication rates with better endothelial preservation	Fungal recurrence, immune rejection	Big-bubble ALK is vital and safe alternative to PK for medically uncontrolled fungal keratitis
Ali et al. [35]	2016–2019	Retrospective cohort	USA	PK: 10,437 EK: 43,567	PK: 113/10437 (1.08%) EK: 74/43567 (0.170%)	None reported	42-day postoperative window	Early postoperative endophthalmitis, severe vision loss risk	Type of keratoplasty, geographic region, CCI	Patients undergoing PK and those with CCI ≥ 3 had higher odds of endophthalmitis relative to EK and those without comorbidities
Chaurasia et al. [36]	2013–2022	Retrospective analysis	India	PK: 16,531 EK: 12,171 ALK: 1356	PK: 6/16531 (0.04%) EK: 33/12171 (0.27%) ALK: 2/1356 (0.14%)	Gram-negative bacteria: *Pseudomonas aeruginosa* *Pseudomonas putida* *Pseudomonas stutzeri* *Enterobacter cloacae* *Serratia marcescens* *Burkholderia mallei* *Klebsiella *spp. *Stenotrophomonas maltophilia* *Proteus mirabilis* Fungal: *Candida* spp. *Scedosporium* spp. *Cladosporium* spp. Gram-positive bacteria: *Staphylococcus aureus* *Enterococcus faecalis* *Corynebacterium striatum* Mixed infection (involving gram-negative, positive, & fungal)	4.5	Donor-related infections	Donor-related infections, multi-drug resistance	Adverse events were of bacterial etiology; whereby gram-negative infections were the majority
Chen et al. [37]	2016–2019	Retrospective cross-sectional	USA	16,726 PK 55,033 EK 71,759 total	PK: 197/16726 (1.18%) EK: 102/55033 (0.185%)	None reported	42-day postoperative window	Higher incidence of endophthalmitis	Older age (85 years), male sex and higher CCI further escalated the risk	Corneal transplant had the highest risk of endophthalmitis than cataract surgery

*PK* penetrating keratoplasty, *EK* endothelial keratoplasty, *ALK* anterior lamellar keratoplasty, *VA* visual acuity, *CCI* Charlson Comorbidity Index

Regarding microbiological culture data and causative microorganisms, all the studies except four [26, 35, 37, 39] reported various causes for post-keratoplasty endophthalmitis (Table 1). In particular, the causative microorganisms of post-keratoplasty endophthalmitis were largely gram-positive bacteria, especially Staphylococcus and Streptococcus species. Gram-negative organisms, particularly Pseudomonas species, were also reported. Fungal pathogens included Candida and Aspergillus species, particularly multidrug-resistant. Fungal infections were also identified, particularly Candida and aspergillus species.

### Methodological quality of the included studies

Most included studies had acceptable methodological quality, with generally low risk of bias across selection and outcome assessment, although comparability should be interpreted cautiously because many studies were non-comparative incidence cohorts. Most examined studies had large sample sizes, adequate follow-up, robust diagnostic methods, and an all-inclusive complication spectrum ≥ Only six studies—Chen et al. [37] (42 days), Ali et al. [35] (42 days), Das et al. [4] (0.75 years), Du et al. [38] (0.5 years), Keyhani et al. [29] (7–14 days), and Guss et al. [21] (0.8 years)—had an inadequate follow-up period compared to the rest. Nevertheless, these studies provided clear incidence rates across different keratoplasty types, making them vital for inclusion in the review. Table 2 provides a summary of methodological quality appraisal outcomes of all included studies.

**Table 2:** Quality assessment of the included studies using the Newcastle–Ottawa Scale

Study ID	Selection				Comparability	Outcome			Total	AHRQ standard
	Cohort representativeness	Exposure ascertainment	Selection of the non-exposed cohort	Demonstration of the outcome of interest	Comparability of cohorts	Assessment of outcome	Adequacy of the follow-up for the outcomes to occur	Adequacy of the follow-up of cohorts		
Guss et al. [21]	★	★	–	★	★	★	★	★	7/9	Good
Leveille et al. [22]	★	★	★	★	★	★	★	★	8/9	Good
Kattan et al. [25]	★	★	–	★	★	★	★	★	7/9	Good
Antonios et al. [23]	★	★	★	★	★	★	★	★	8/9	Good
Cameron et al. [24]	★	★	★	★	★	★	★	★	8/9	Good
Aiello et al. [26]	★	★	–	★	★★	★	★	–	7/9	Good
Aaberg et al. [27]	★	★	–	★	★	★	★	–	6/9	Good
Eifrig et al. [28]	★	★	–	★	★	★	★	–	6/9	Good
Keyhani et al. [29]	★	★	★	★	★	★	–	★	7/9	Good
Jambulingam et al. [3]	★	★	★	★	★	★	★	–	7/9	Good
Wykoff et al. [30]	★	★	★	★	★	★	★	★	8/9	Good
Du et al. [38]	★	★	★	★	★★	–	★	★	8/9	Good
Chen et al. [32]	★	★	★	★	★★	–	★	★	8/9	Good
Park et al. [31]	★	★	★	★	★	★	★	★	8/9	Good
Borkar et al. [33]	★	★	★	★	★	★	★	★	7/9	Good
Das et al. [4]	★	★	★	★	★	★	★	★	8/9	Good
Anshu et al. [39]	★	★	★	★	★★	★	★	★	9/9	Good
Chen et al. [34]	★	★	–	★	★	★	★	–	6/9	Good
Ali et al. [35]	★	★	★	★	★★	★	–	★	8/9	Good
Chaurasia et al. [36]	★	★	★	★	★	★	★	★	8/9	Good
Chen et al. [37]	★	★	★	★	★★	★	–	★	8/9	Good

*AHRQ* agency for healthcare research and quality

### Pooled incidence of Infectious endophthalmitis

**Fig. 2:**
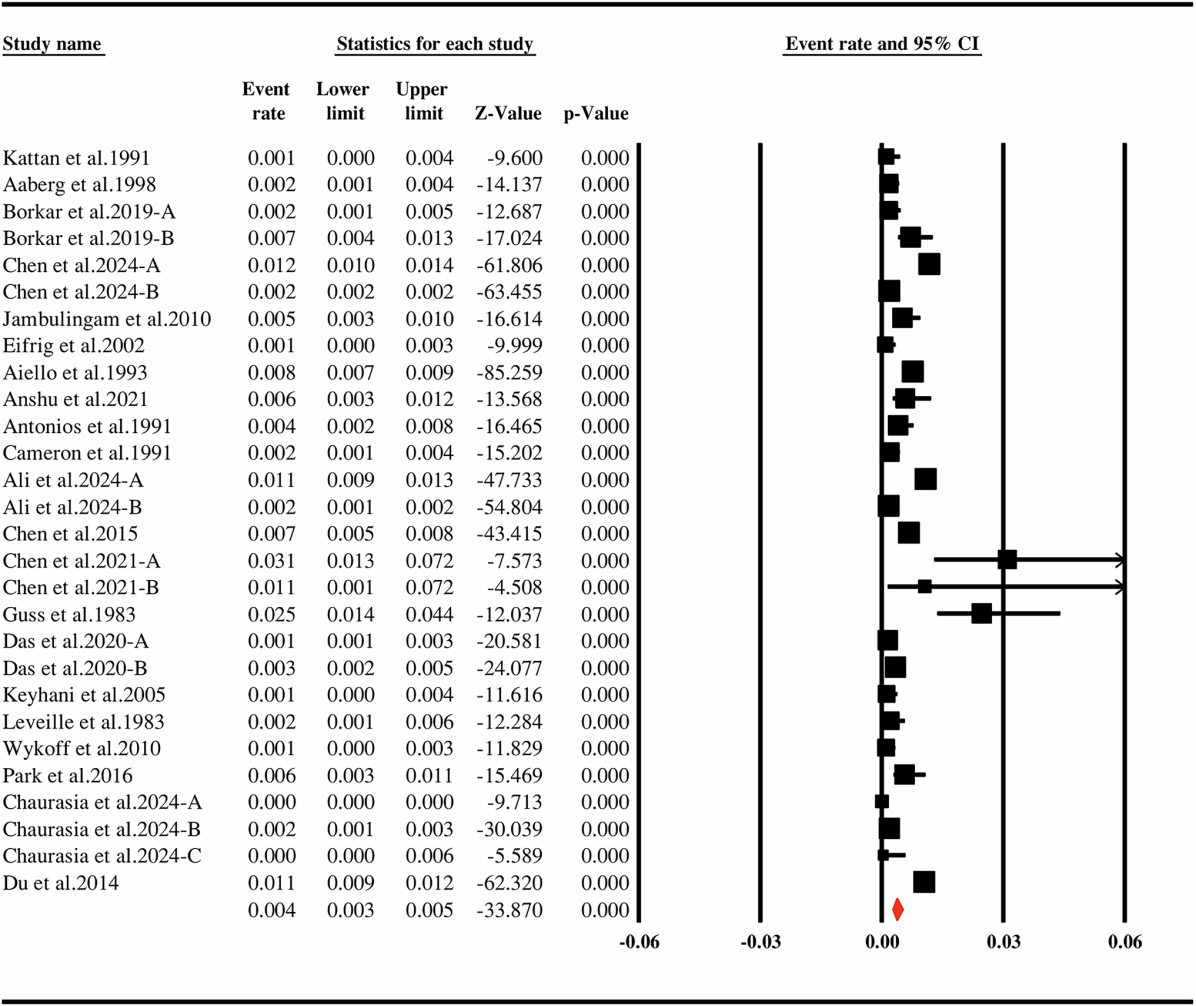
Forest plot of the pooled incidence of infectious endophthalmitis after keratoplasty using a random-effects single-arm meta-analysis

A random-effects meta-analysis of incidence proportions from the twenty-one included studies revealed that the pooled incidence of infectious endophthalmitis after keratoplasty was 0.40% (95% CI 0.30–0.50) (Fig. 2), with study-level incidence ranging from 0.10 [25] to 3.10% [34] across the studies. The corresponding prediction interval ranged from 0.08% to 1.95%, indicating substantial between-study variability in the expected incidence across different settings. Substantial heterogeneity was observed across the studies (I^2^ = 95.75%, τ ^2^ = 0.59, p < 0.001), which justified the use of a random-effects model.

### Subgroup analyses

**Fig. 3:**
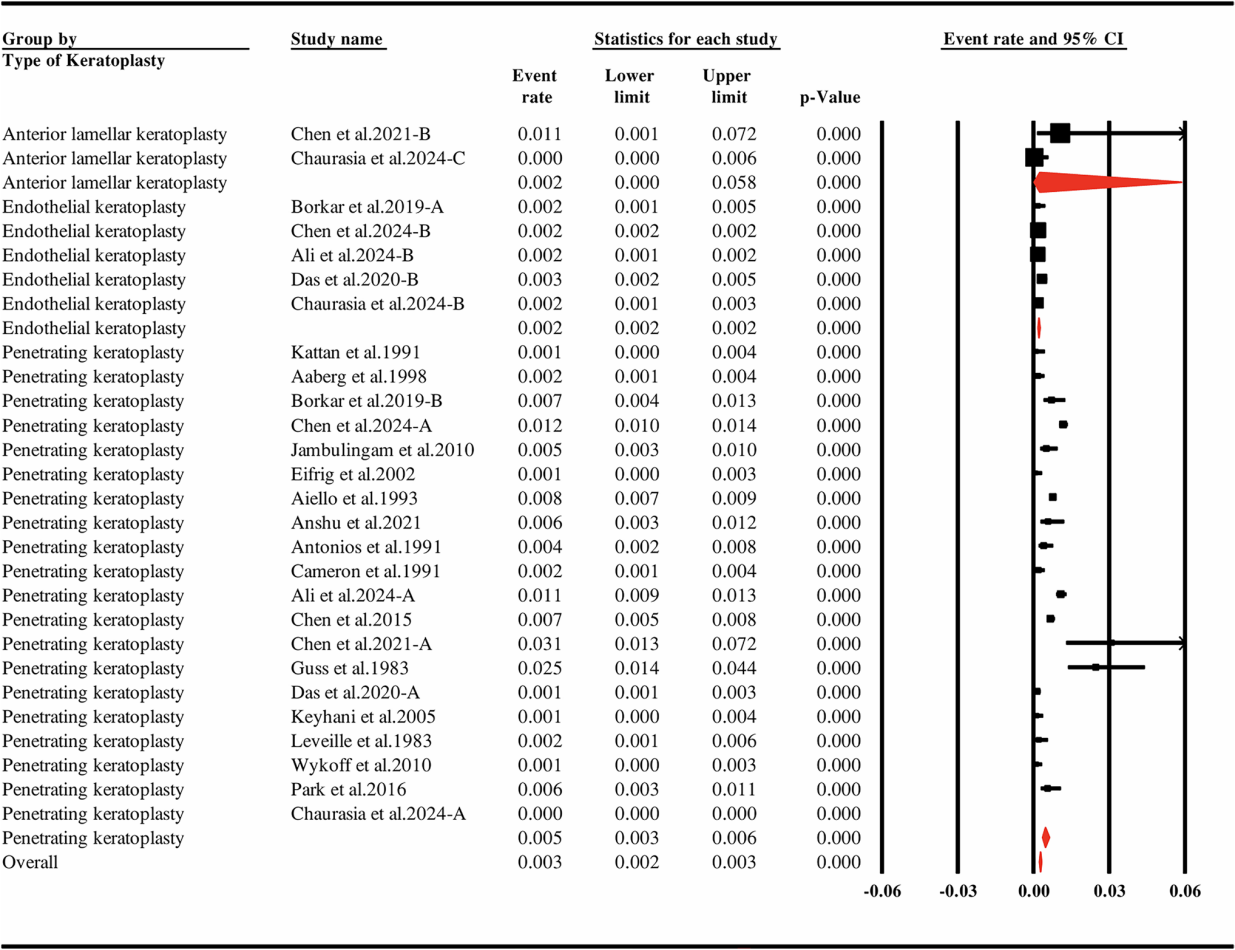
Forest plot of the pooled incidence of infectious endophthalmitis stratified by keratoplasty type, including penetrating keratoplasty, endothelial keratoplasty, and anterior lamellar keratoplasty

Subgroup analysis revealed that the pooled incidence of endophthalmitis varied according to the type of keratoplasty surgery. The pooled incidence following PK was 0.50% (95% CI 0.30–0.60), whereas studies focusing on ALK reported a pooled incidence of 0.20% (95% CI 0.01–0.06) and those focusing on EK reported a pooled incidence of 0.20% (95% CI 0.20–0.30) (Fig. 3) ; these subgroup differences should be interpreted descriptively because they were derived from pooled single-arm incidence estimates. The test for subgroup differences was statistically significant (p < 0.0001). Corresponding PIs for PK and EK were 0.1–1.5% and 0.08–0.51%, respectively, demonstrating variation in incidences across studies. Both PK and EK had higher heterogeneity—I^2^ = 90.34%, τ ^2^ = 0.30, p < 0.001 and I^2^ = 88.34%, τ ^2^ = 0.02, p = 0.130, respectively.

**Fig. 4:**
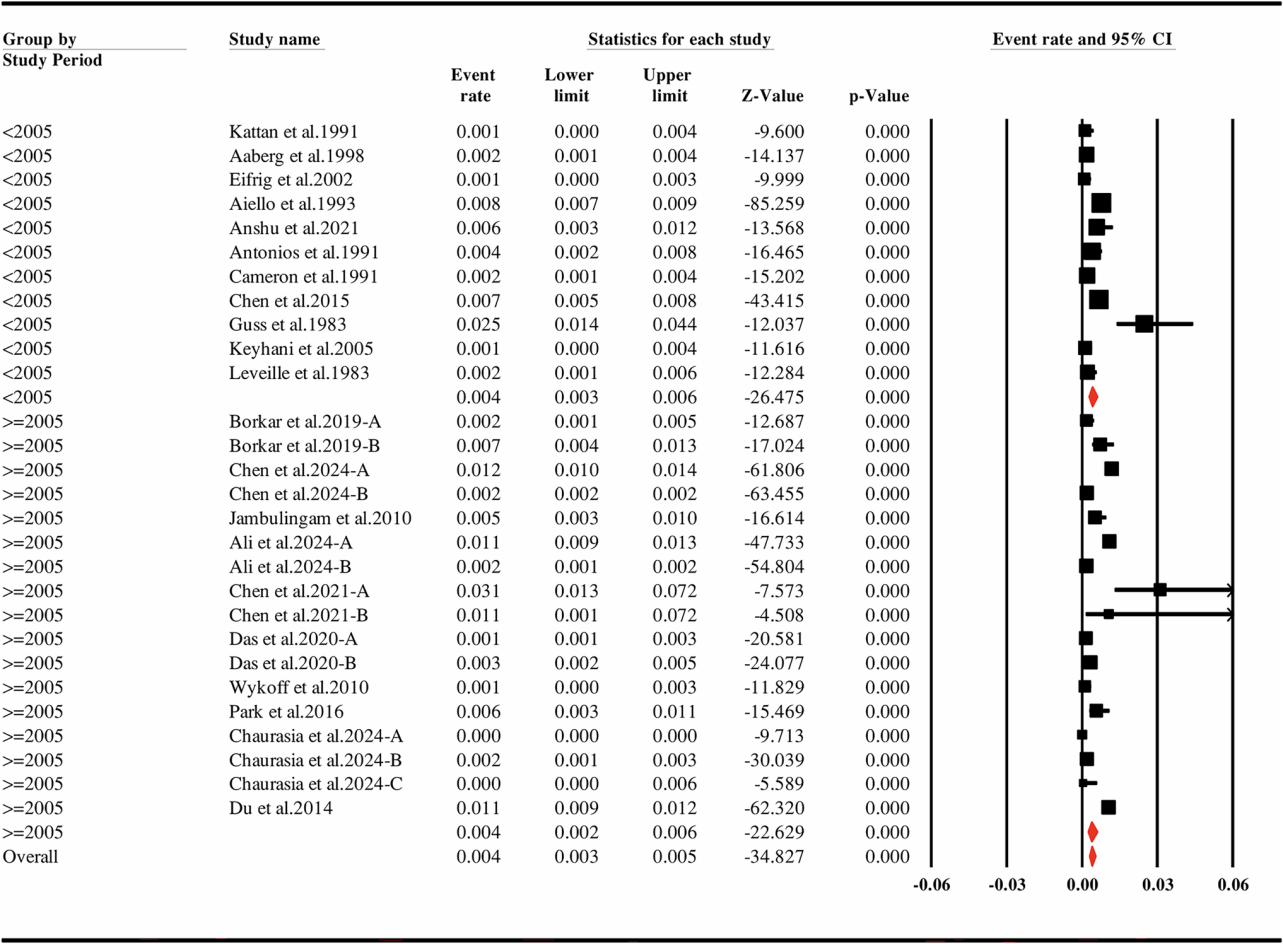
Forest plot of the pooled incidence of infectious endophthalmitis stratified by study period (pre-2005 vs. post-2005)

To examine the temporal trends in the incidence of infectious endophthalmitis after keratoplasty, we performed subgroup analyses according to the study period. Studies were categorized into pre- and post-2005 subgroups. The pooled analysis demonstrated a similar incidence in studies conducted before 2005 (0.40%; 95% CI 0.30–0.6) and those conducted after 2005 (0.40%; 95% CI 0.20–0.60) (Fig. 4). The difference between these subgroups was not statistically significant (p = 0.71). The PI for pre-2005 was 0.10 to 1.53%, while post-2005 was 0.05 to 2.95%. Heterogeneity for both subgroups was substantially high, with I^2^ = 86.27%, τ ^2^ = 0.32, p < 0.001 for pre-2005 and I^2^ = 97.03%, τ ^2^ = 0.86, p < 0.001 for post-2005.

**Fig. 5:**
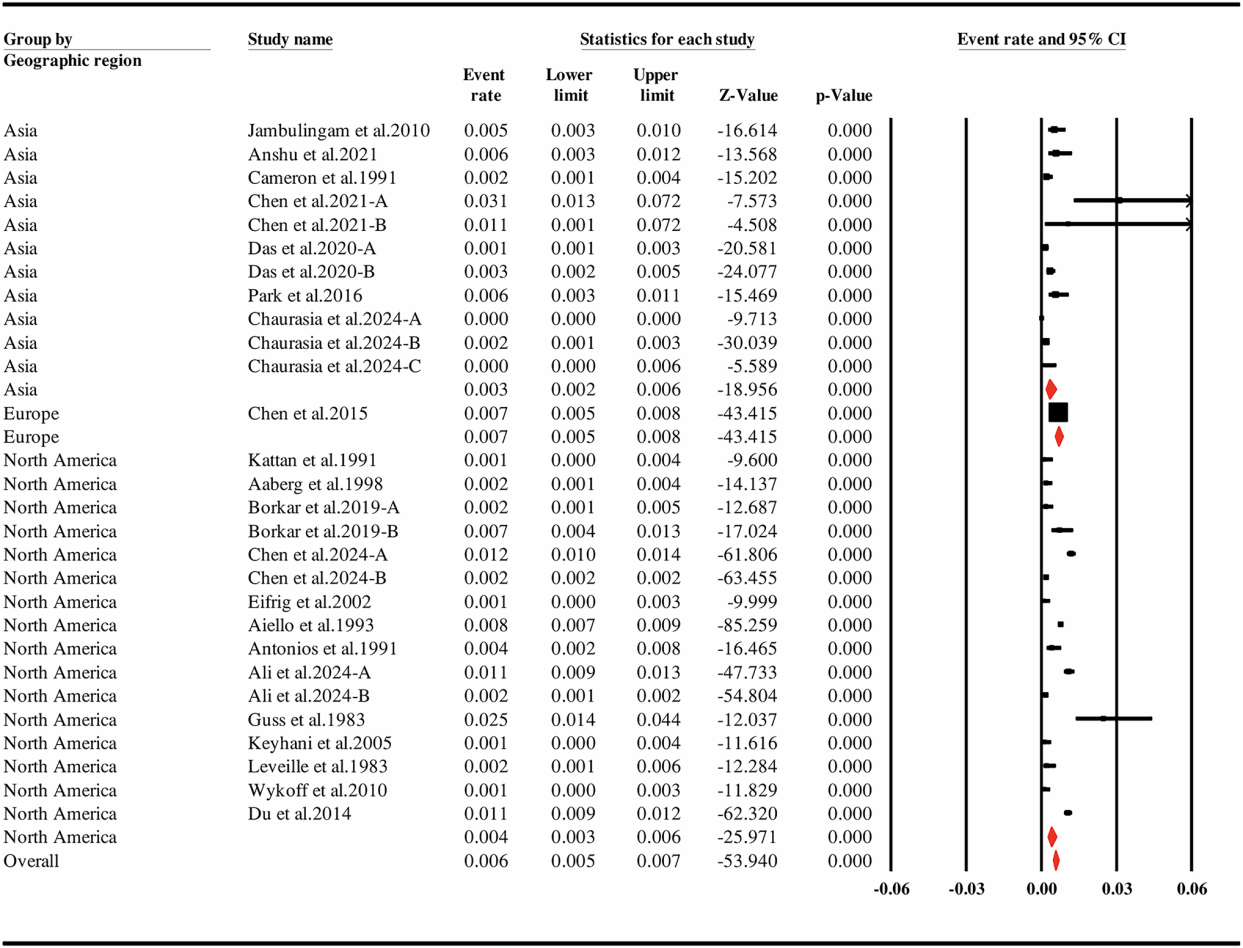
Forest plot of the pooled incidence of infectious endophthalmitis stratified by geographic region (North America, Asia, and Europe)

When subgroup analysis was performed based on the geographic region, we observed that the incidence of infectious endophthalmitis was 0.40% (95% CI 0.30–0.60) in North America, 0.30% (95% CI 0.20–0.60) in Asia, and 0.7% (95% CI 0.50–0.80) in Europe (Fig. 5). The test for subgroup differences was statistically significant (p = 0.007). Corresponding PIs for Asia and North America were 0.04–2.41% and 0.07–2.19%, respectively. The subgroup analysis demonstrated high variation of incidences, with substantial heterogeneity of I^2^ = 84.98%, τ ^2^ = 0.74, p < 0.001 for Asia and I^2^ = 97.11%, τ ^2^ = 0.60, p < 0.001 for North America.

**Fig. 6:**
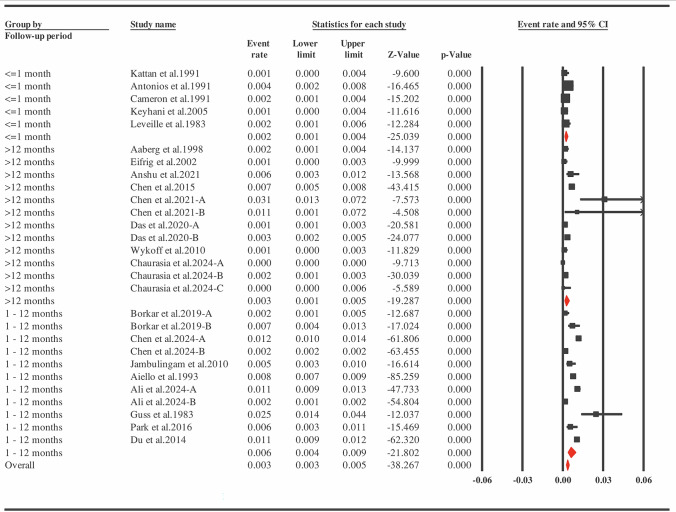
Forest plot of the pooled incidence of infectious endophthalmitis stratified by follow-up duration (≤ 1 month, 1–12 months, and > 12 months)

Subgroup analyses were also conducted according to different follow-up durations (≤ 1 month, 1–12 months, and > 12 months). The statistical analyses showed that the pooled incidence of endophthalmitis was 0.20% (95% CI 0.10–0.40) for studies with ≤ 1 month follow-up, 0.60% (95% CI 0.40–0.90) for studies with 1–12 months follow-up, and 0.30% (95% CI 0.10–0.50) for studies with > 12 months follow-up (Fig. 6). The test for subgroup differences was statistically significant (p = 0.007). Corresponding PI for ≤ 1 month were 0.05–0.82%, 0.11–3.33% for 1–12 months, and 0.04–2.46% for > 12 months. The subgroup analysis demonstrated variation of heterogeneity across different follow-up durations, I^2^ = 24.81%, τ ^2^ = 0.07, p = 0.256 for ≤ 1 month, I^2^ = 97.86%, τ ^2^ = 0.55, p < 0.001 for 1–12 months, and I^2^ = 89.02%, τ ^2^ = 0.80, p < 0.001 for < 12 months.

### Risk factors

The most consistently identified risk factor across studies was the type of keratoplasty, with PK associated with significantly higher pooled incidence estimates of endophthalmitis compared to EK or ALK [35, 40]. Similarly, Du et al. documented that cataract surgery carried significantly lower risk of endophthalmitis than corneal transplant [38]. Other risk factors included socioeconomic status (low-income subsidy was associated with higher risk) [38], aphakia [21, 22], immunosuppressant use [38], and anterior vitrectomy [21, 26, 40]. Systemic health also played a role; a Charlson Comorbidity Index (CCI) ≥ three or more was associated with increased risk [35, 37]. Geographic location also influenced the incidence of endophthalmitis, with practices in the South of the United States reporting higher rates than those in the Midwest, West, or Northeast [35]. Donor-related factors were also significant: infections in donors and fungal contamination of donor rims were strongly associated with infection [22–24, 27, 29].

Moreover, only a few of the included studies highlighted age as a risk factor for post-keratoplasty endophthalmitis or graft outcomes [35, 37–39]. Du et al. noted that older patients (≥ 75) had significantly higher risk of endophthalmitis than their counterparts (65–74) [38]. Ali and colleagues analyzed outcomes according to age groups (i.e., 65 to 74, 75 to 84, and ≥85 years, but there was no significant association between incidence of endophthalmitis and patient age [35]. Anshu and co-authors noted that younger donor age was substantially associated with better long-term graft survival [39]. Conversely, Chen et al. highlighted that age age was directly associated with endophthalmitis risk [37]. Specifically, older patients (75–84 year) had high risk (OR 1.09), while those aged ≥ 85 had a higher risk (OR 1.36) of endophthalmitis post-keratoplasty.

### Complications related to keratoplasty

Keratoplasty was also associated with different complications, as reported by the included studies. Some of the reported complications were poor visual outcomes, retinal detachment, severe visual loss/morbidity, graft infection, phthisis bulbi, corneal graft edema, risk of glaucoma, wound dehiscence, and donor-related infections. Visual outcomes following endophthalmitis were generally poor across studies. Chen et al. [32] reported a 5-year graft survival rate of only 27% in patients who developed endophthalmitis, with a mean best-corrected visual acuity (BCVA) of 1.13 logMAR. Borkar et al. [40] found significantly worse visual outcomes and higher graft failure rates in PK-related infections compared to EK. Das et al. [4] noted that only 39.1% of eyes achieved vision ≥ 20/200, though globe salvage was successful in 85.7% of cases.

### Publication bias

**Fig. 7:**
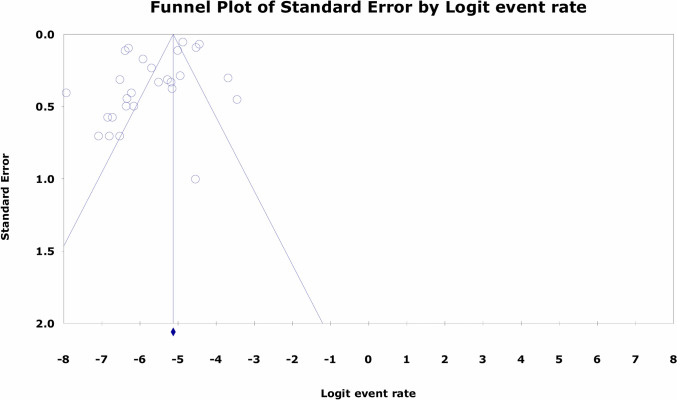
Funnel plot assessing potential small-study effects and asymmetry among the included studies in the meta-analysis.

The funnel plot generated from the included studies presents a visual assessment of potential small-study effects and asymmetry (Fig. 7). In this plot, the event rates are plotted against study precision (1/standard error). Ideally, in the absence of small-study effects, the studies should be distributed symmetrically around the mean effect size, forming an inverted funnel shape. However, the distribution in the current plot appears slightly asymmetrical, with a noticeable clustering of studies on one side of the logit event rate. This visual pattern may suggest the presence of small-study effects, potentially reflecting differences in study size, heterogeneity, or reporting. As a result, we performed Egger's test to confirm the significance of publication bias. The Egger's regression test indicated a significant intercept (Intercept: -2.86, P = 0.04), suggesting the presence of publication bias.

### Sensitivity analysis and meta-regression

**Fig. 8:**
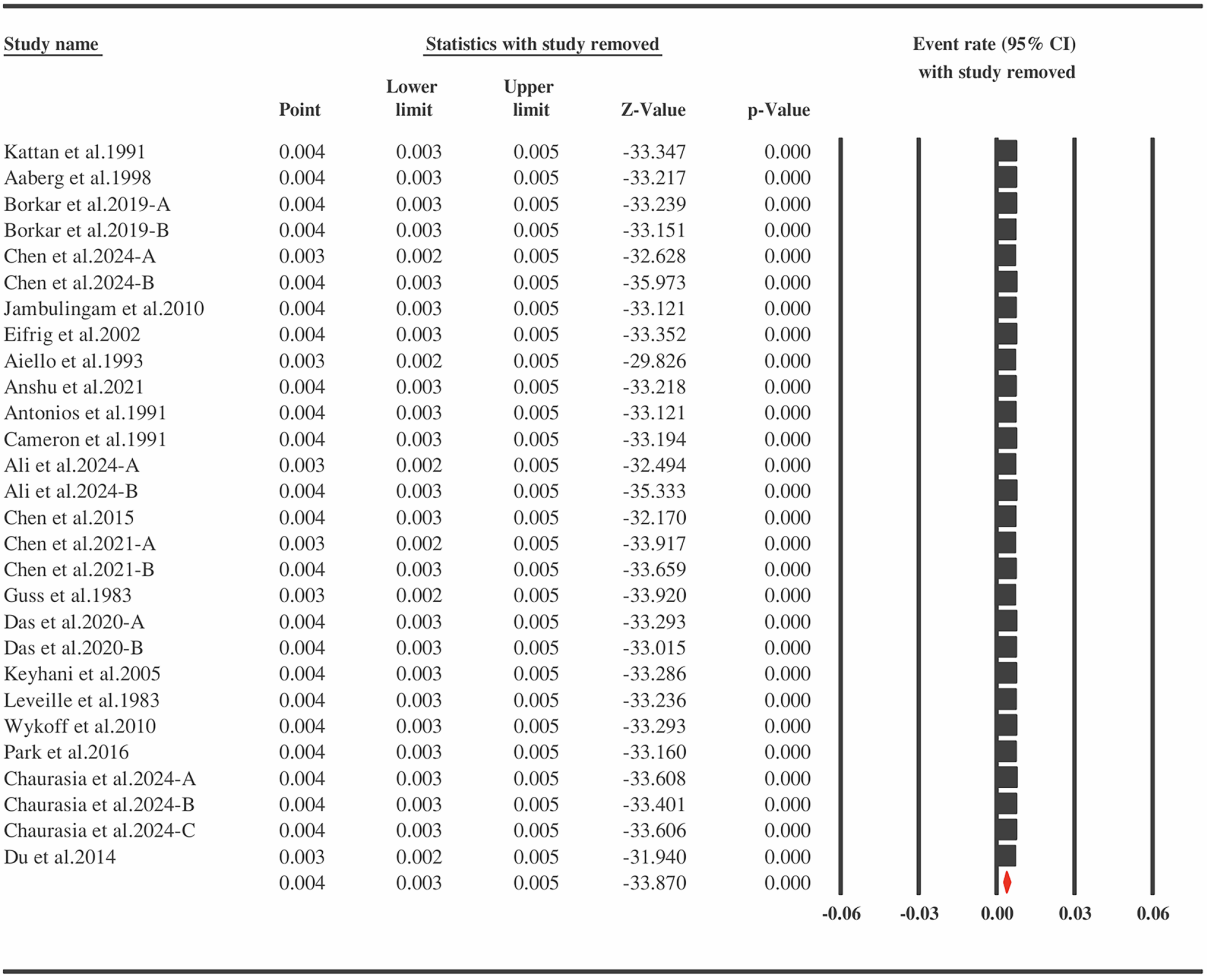
Leave-one-out sensitivity analysis showing the influence of sequential exclusion of individual studies on the pooled incidence estimate of infectious endophthalmitis after keratoplasty

A leave-one-out sensitivity analysis was conducted to determine the influence of each study on the pooled estimate. Exclusion of one study simultaneously did not result in any major change in the overall pooled estimate (Fig. 8).

Conversely, a meta-regression was performed to identify the source of heterogeneity. From the analysis, the type of keratoplasty, study period (< 2005 or > 2005), and geographic region did not have any significance in contributing to sources of heterogeneity in the statistical analysis (Table 3). However, follow-up period among included studies were identified to the significant source of heterogeneity (p = 0.0012).

**Table 3:** A meta-regression to determine the sources of heterogeneity

Variable	Coefficient	Standard error	95% CI	P value
Type of keratoplasty	0.48	0.84	−1.17–2.13	0.054
Study period	0.36	0.37	−0.36–1.08	0.331
Geographic region	-0.17	0.33	−0.82–0.47	0.598
Follow-up period	1.58	0.49	0.62–2.53	0.0012

## Discussion

Our systematic review and meta-analysis provide an updated and comprehensive evaluation of the incidence and risk factors of infectious endophthalmitis following keratoplasty, incorporating data from PK, EK, and ALK surgical techniques. The overall pooled incidence of infectious endophthalmitis after keratoplasty was found to be 0.40% (random effects model, 95% CI 0.30–0.50), which, although low, underscores a persistent clinical risk. This updated incidence estimate is comparable to the overall pooled estimate reported by Taban et al. (0.382%), while exceeding the 0.200% rate reported for the early-2000s subgroup Notably, the inclusion of more recent data in our analysis reflects changing surgical practices and may explain the observed differences.

Focusing on PK, the pooled incidence was estimated at 0.50%, reinforcing the notion that PK carries a higher risk of postoperative endophthalmitis compared to other keratoplasty techniques. These findings are consistent with the results reported by Chen et al., who reported a PK-related incidence of 0.67%, while Keyhani et al. (2005) found an even higher rate of 0.92%, particularly associated with fungal contamination from donor tissue [29, 32]. Similarly, Borkar et al. (2019) and Alharbi et al. (2013) reported PK incidence rates of 0.7% and 0.68%, respectively, both identifying PK as more susceptible to infection than EK or lamellar approaches [40, 41]. These findings corroborate the pooled data and emphasize the ongoing risk despite advancements in donor screening and surgical protocols.

When stratifying by technique, Our comparison between PK and EK found a trend toward higher pooled incidence estimates of endophthalmitis with PK. Individual studies such as Ali et al. and Borkar et al. demonstrated significantly higher odds of endophthalmitis with PK (ORs of 5.21 and 3.30, respectively), supporting the argument for transitioning to lamellar techniques where appropriate [35, 40]. In contrast, Das et al. uniquely reported a higher incidence in EK than PK (0.34% vs. 0.15%), attributing this to multidrug-resistant gram-negative organisms, especially Pseudomonas species, underscoring regional microbiological variations [3]. Research evidence shows that PKs are likely to have higher rates of endophthalmitis than EKs due to the inherent variations in surgical techniques including exposure of sutures and larger circumferential wound in PK [35]. Song et al. [42] note that the differences in post-keratoplasty incidences of endophthalmitis are attributed to variation in graft techniques. The authors note that utilization of lamellar approaches might minimize some infection pathways compared to full-thickness procedures, leading to low rates of endophthalmitis [42]. Besides high incidence of endophthalmitis, PKs bring higher risks, such as loose epithelium and secondary wound dehiscence, than lamellar keratoplasties [43].

Several risk factors emerged consistently across studies, including donor-related issues such as infectious cause of death, positive fungal rim cultures, and late suture removal [29, 32, 44]. Patient-level risks such as high Charlson Comorbidity Index (CCI ≥ 3), as noted by Ali et al. (2024), and procedural factors like anterior vitrectomy also contributed to increased infection risk [35]. Besides, gram-positive organisms were the predominant microbiological spectrum, with occasional gram-negative and fungal pathogens. These results demonstrate how surgical technique and donor and recipient characteristics have a role in the multifactorial nature of post-keratoplasty infections.

Our analysis showed that endophthalmitis incidence was higher in Europe compared to Asia and North America. Post-keratoplasty infectious endophthalmitis is more likely influenced by procedure-related, donor-related, and microbiological factors, although recipient immunological status may also contribute in selected cases. Literature evidence indicates that the causative organisms of endophthalmitis vary by geographic region [45], which may contribute to variation in incidences in Asia, North America, and Europe.

Our results also showed that PK, EK, and ALK were associated with different complications. Major reported complications were poor visual outcomes, retinal detachment, and severe visual loss/morbidity. Visual outcomes following post-keratoplasty endophthalmitis were generally poor across studies. Our findings align with Turnbull et al.'s review, which suggests that even though EK has progressively developed, fewer patients than expected attain the best corrected visual acuity of 20/20 [46]. In particular, Park and colleagues note that vitreoretinal complications after keratoplasty significantly escalated among patients, contributing to persistent reduction of corrected distance visual acuity [31]. Literature evidence shows that such poor visual outcomes are observed despite healthy grafts and the absence of ocular comorbidities [47].

Future studies should establish the correlation between visual quality and contributing factors following keratoplasty to optimize patient-reported and visual outcomes while enhancing surgical safety and efficacy. Additionally, most of the included studies in our review are retrospective, without the standardization assurance accorded by prospective trials. Even though these retrospective studies offer useful statistics on the incidence of endophthalmitis, they barely enable precise direct comparison of different types of keratoplasties. Future prospective studies and registry-based comparative analyses will be valuable in allowing a more accurate comparison of different keratoplasty techniques with respect to the incidence of endophthalmitis.

Overall, high heterogeneity was observed in our statistical analysis. This high variation of outcomes among included studies reflects the difference in study periods, patient populations, follow-up durations, and keratoplasty surgical techniques. We utilized the random-effects model to ensure conservative pooled statistical outcomes, which account for methodological variation.

### Strengths and limitations

This systematic review and meta-analysis comprehensively evaluated 21 studies in assessing the incidence of endophthalmitis. We included cohort observational studies that were methodologically robust with good quality, reducing the risk of bias in the pooled estimates. Despite these strengths, our review has several significant limitations. A major issue was the high degree of heterogeneity among included studies, driven by differences in study populations, surgical techniques, diagnostic criteria, and follow-up durations, which limits the generalizability of the pooled estimates. Excluding studies based on language may have introduced selection bias, leading to the exclusion of studies with endophthalmitis incidences. The limited number of studies directly comparing PK and EK also reduced the statistical power of subgroup analyses, making cross-group interpretations tentative. Most included studies were retrospective or observational, prone to selection bias and inconsistent reporting, with variable or unclear diagnostic criteria for endophthalmitis.

Additionally, underreporting of infection events, especially in studies with short follow-up or zero-event corrections, may have led to imprecise incidence estimates. Variability in follow-up duration further complicates comparisons across studies because it affects the time available for event capture. Geographic and institutional differences in surgical practices, donor tissue handling, and infection control protocols may also affect results and limit their applicability to other settings. The lack of individual patient-level data prevented adjustment for essential risk factors, such as age or immune status.

## Conclusion

This systematic review and meta-analysis highlight that infectious endophthalmitis remains a rare but serious complication following corneal transplantation, with PK demonstrated higher pooled incidence estimates compared to ALK or EK in this synthesis. While the overall risk remains low, significant variability exists across studies due to differences in surgical techniques, patient characteristics, and postoperative care. Identified risk factors, such as combined procedures, donor-related contamination, and patient comorbidities, emphasize the need for rigorous perioperative protocols. The microbial spectrum was dominated by gram-positive organisms, though resistant gram-negative and fungal pathogens were also implicated. Visual outcomes following endophthalmitis were generally poor, underscoring the clinical importance of prevention. Despite the heterogeneity in reported data, our findings suggest that EK may be associated with a lower pooled incidence of infectious endophthalmitis. Further large-scale, standardized studies are warranted to validate these trends and refine preventive strategies.

## Supplementary Information

Below is the link to the electronic supplementary material.

Supplementary file1 (DOCX 23 kb)

## Data Availability

All data generated or analyzed in this study are fully contained within the main manuscript, its tables and figures, and the supplementary materials. No additional unpublished datasets are available.

## Declarations

### Guarantor

C.-M. C. (Chi-Ming Chan) is the guarantor of this work and accepts full responsibility for the integrity of the study, the accuracy of the data analysis, and the decision to submit the manuscript for publication. The guarantor had full access to all extracted data and takes responsibility for the overall content of the manuscript.
