# Supplementary material for: Incidence of infectious endophthalmitis after keratoplasty surgery: an updated systematic review and meta-analysis
Source: Infection. 2026 Apr 17;54(4):1701–23. doi: 10.1007/s15010-026-02772-4 (PMC13476306; doi:10.1007/s15010-026-02772-4)
Supplement: Supplementary file 1 [file 15010_2026_2772_MOESM1_ESM.docx]

Table S 1: Full search strategy for each database

| Database | Search query | Results |
| --- | --- | --- |
| PubMed | ("endophthalmitis"[MeSH Terms] OR "endophthalmitis"[All Fields] OR "endophthalmitides"[All Fields]) AND ("corneal transplantation"[MeSH Terms] OR ("corneal"[All Fields] AND "transplantation"[All Fields]) OR "corneal transplantation"[All Fields] OR "keratoplasties"[All Fields] OR "keratoplasty"[All Fields]) | 626 |
| Science Direct | (endophthalmitis) AND ("penetrating keratoplasty" OR "endothelial keratoplasty" OR "corneal transplant" OR "corneal transplantation") | 276 |
| Web of Science | "endophthalmitis" AND ("penetrating keratoplasty" OR "endothelial keratoplasty" OR "corneal transplant") | 127 |
| Cochrane Library | Keratoplasty [MESH] AND Endophthalmitis [MESH] OR ((endophthalmitis):ti,ab,kw AND ("penetrating keratoplasty" OR "endothelial keratoplasty" OR "corneal transplant")):ti,ab,kw | 209 |
| Scopus | TITLE-ABSTRACT-KEYWORD (endophthalmitis)  AND  TITLE-ABSTRACT-KEYWORD ("penetrating keratoplasty" OR "corneal transplant" OR "corneal transplantation") | 261 |

Note: ti – title; ab – abstract; kw – keywords

Table S2: Exclusion reasons for 43 articles identified through database search and other methods

| Exclusion reason | Number of articles excluded | Author ID (Links) |
| --- | --- | --- |
| Focused on animal subjects. Did not evaluate humans as the participants | 4 | Yang et al. 2020 (<https://pubmed.ncbi.nlm.nih.gov/32754262/>)  Kishimoto et al. 2022 (<https://pubmed.ncbi.nlm.nih.gov/35363260/>)  Tsuchiya et al. 2008 (<https://pubmed.ncbi.nlm.nih.gov/18853321/>)  Schrage et al. 2014 (<https://pubmed.ncbi.nlm.nih.gov/23765372/>) |
| Lacked endophthalmitis rates | 15 | Polack 1984 (<https://pubmed.ncbi.nlm.nih.gov/6399230/>) Kuhn et al. 2023 (<https://pubmed.ncbi.nlm.nih.gov/36468294/>)  Meredith 1999 (<https://pubmed.ncbi.nlm.nih.gov/10206582/>)  Cunningham et al. 2018 (<https://pubmed.ncbi.nlm.nih.gov/29768116/>)  Kemer et al. 2021 (<https://pubmed.ncbi.nlm.nih.gov/34963266/>)  Al-Lozi et al. 2022 (<https://pubmed.ncbi.nlm.nih.gov/33426987/>)  Visliseli et al. 2017 (<https://pubmed.ncbi.nlm.nih.gov/27817919/>)  Zapp et al. 2018 (<https://pubmed.ncbi.nlm.nih.gov/29724209/>)  Sridhar et al. 2015 (<https://pubmed.ncbi.nlm.nih.gov/25658178/>)  Wang et al. 2016 (<https://pubmed.ncbi.nlm.nih.gov/27434905/>)  Abouammoh et al. 2025 (<https://pubmed.ncbi.nlm.nih.gov/41213074/>)  Soni et al. 2022 (<https://pubmed.ncbi.nlm.nih.gov/35369775/>)  Lam et al. 1998 (<https://pubmed.ncbi.nlm.nih.gov/9614531/>)  Foroutan et al. 2009 (<https://pubmed.ncbi.nlm.nih.gov/19730092/>)  Ritterband et al. 2002 (<https://pubmed.ncbi.nlm.nih.gov/11812434/>) |
| Did not evaluate keratoplasty surgery | 6 | Mamalis 2019 (<https://pubmed.ncbi.nlm.nih.gov/31470938/>)  Morris et al. 2021 (<https://pubmed.ncbi.nlm.nih.gov/34075812/>)  Behrens 2008 (<https://pubmed.ncbi.nlm.nih.gov/19016197/>)  Forster 1995 (<https://pubmed.ncbi.nlm.nih.gov/7487626/>)  Honavar 2022 (<https://pubmed.ncbi.nlm.nih.gov/35086194/>)  Palestine et al. 2020 (<https://pubmed.ncbi.nlm.nih.gov/32507272/>) |
| Irrelevant outcomes | 18 | Soiberman et al. 2023 (<https://pubmed.ncbi.nlm.nih.gov/37890881/>)  Huang 2007 (<https://pubmed.ncbi.nlm.nih.gov/17307675/>)  Patel 2011 (<https://pubmed.ncbi.nlm.nih.gov/21335107/>)  Aggarwal 1997 (<https://pubmed.ncbi.nlm.nih.gov/9135378/>)  Price et al. 2011 (<https://pubmed.ncbi.nlm.nih.gov/21251490/>)  Smit et al. 2024 (<https://pubmed.ncbi.nlm.nih.gov/39102496/>)  Flynn 2001 (<https://pubmed.ncbi.nlm.nih.gov/11756878/>)  Werner 2021 (<https://pubmed.ncbi.nlm.nih.gov/34468445/>)  Januschowski 2021 (<https://pubmed.ncbi.nlm.nih.gov/33666728/>)  Doft 1991 (<https://pubmed.ncbi.nlm.nih.gov/2012545/>)  Oslon 2021 (<https://pubmed.ncbi.nlm.nih.gov/34173382/>)  Vanathi 2024 (<https://pubmed.ncbi.nlm.nih.gov/38131560/>)  Medsinge 2013 (<https://pubmed.ncbi.nlm.nih.gov/24049121/>)  Malik et al. 2004 (<https://pubmed.ncbi.nlm.nih.gov/15068425/>)  Panthagani et al. 2020 (<https://pubmed.ncbi.nlm.nih.gov/32296129/>)  Sharma et al. 2020 (<https://pubmed.ncbi.nlm.nih.gov/32305350/>)  Singh et al. 2019 (<https://pubmed.ncbi.nlm.nih.gov/31571625/>)  Boychev et al. 2023 (<https://pubmed.ncbi.nlm.nih.gov/36633636/>) |
| Did not meet any of the inclusion criteria | 16 | Robbins et al. 2021 (<https://pubmed.ncbi.nlm.nih.gov/32949491/>)  Alharbi et al. 2021 (<https://pubmed.ncbi.nlm.nih.gov/24131044/>)  Dave et al. 2019 (<https://pubmed.ncbi.nlm.nih.gov/30073760/>)  Kunimoto et al. 2004 (<https://pubmed.ncbi.nlm.nih.gov/14962427/>)  Dave et al. 2020 (<https://pubmed.ncbi.nlm.nih.gov/32709783/>)  Velez et al. 2023 (<https://pubmed.ncbi.nlm.nih.gov/36441843/>)  Fathai et al. 2023 (<https://pubmed.ncbi.nlm.nih.gov/37817122/>)  Mgboji et al. 2023 (<https://pubmed.ncbi.nlm.nih.gov/37146289/>)  Liu et al. 2015 (<https://pubmed.ncbi.nlm.nih.gov/25633311/>)  Bor et al. 2011 (<https://pubmed.ncbi.nlm.nih.gov/21563747/>)  Alsarhani et al. 2023 (<https://pubmed.ncbi.nlm.nih.gov/35404731/>)  Wong et al. 2023 (<https://pubmed.ncbi.nlm.nih.gov/36731080/>)  Dave et al. 2023 (<https://pubmed.ncbi.nlm.nih.gov/34812082/>)  Maguire 2008 (<https://pubmed.ncbi.nlm.nih.gov/18356929/>)  Panchal et al. 2019 (<https://pubmed.ncbi.nlm.nih.gov/30879381/>)  Tran et al. 2018 (<https://pubmed.ncbi.nlm.nih.gov/29468222/>) |
